# Structural characteristics of the SARS-CoV-2 Omicron lineages BA.1 and BA.2 virions

**DOI:** 10.1038/s41392-023-01385-9

**Published:** 2023-03-20

**Authors:** Xiaoyu Ma, Yanqun Wang, Yuanzhu Gao, Yiliang Wang, An Yan, Jiantao Chen, Lu Zhang, Peiyi Wang, Jincun Zhao, Zheng Liu

**Affiliations:** 1 Cryo-electron Microscopy Center, Southern University of Science and Technology, Shenzhen, Guangdong China; 2 State Key Laboratory of Respiratory Disease, National Clinical Research Center for Respiratory Disease, Guangzhou Institute of Respiratory Health, the First Affiliated Hospital of Guangzhou Medical University, Guangzhou, Guangdong China; 3 Health and Quarantine Laboratory, Guangzhou Customs District Technology Centre, Guangzhou, China; 4 Guangzhou Laboratory, Bio-Island, Guangzhou, China; 5 Institute of Infectious disease, Guangzhou Eighth People’s Hospital of Guangzhou Medical University, Guangzhou, Guangdong, China

**Keywords:** Structural biology, Infectious diseases

**Dear Editor**,

Variants of concern (VOCs) of SARS-CoV-2 have increased transmissibility, virulence, and resistance. The emergence of new VOCs continues to pose great challenges to public health. The detailed structures of the intact Omicron viruses remain unveiled. In this study, we report the architecture of both Omicron BA.1 and BA.2 lineages using cryo-electron tomography (cryo-ET). The sub-tomogram averaging of viral spikes, in both pre-fusion and post-fusion conformations are also investigated.

We isolated authentic Omicron subvariants from nasopharyngeal swabs of COVID-19 patients and propagated them in Vero E6 cells. For cryo-ET, BA.1 and BA.2 virions were fixed with paraformaldehyde. Direct visualization of the intact virions shows typical features of a coronavirus: a spherical particle containing a dense viroplasm, bounded by a lipid bilayer with spike protrusions (Fig. 1a, b, e, f, Supplementary Movie 1 and 2). Virions from both Omicron BA.1 and BA.2 display a uniform spherical shape, with enveloped long/short axis ratio close to 1.0 (1.0 ± 0.02 for BA.1 and 1.0 ± 0.02 for BA.2, Fig. 1d, h). In contrast, the wild type (WT) SARS-CoV-2 virions are oblate spheroidal.^1^ The mean diameter of Omicron BA.1 was 75.0 ± 7.9 (mean ± SD) nm, slightly smaller than BA.2, which was measured as 81.5 ± 6.6 nm (Fig. 1c, g). Both BA.1 and BA.2 have a similar size to the reported diameter of WT SARS-CoV-2 virions that propagated in the same Vero cells.^1,2^

**Fig. 1:**
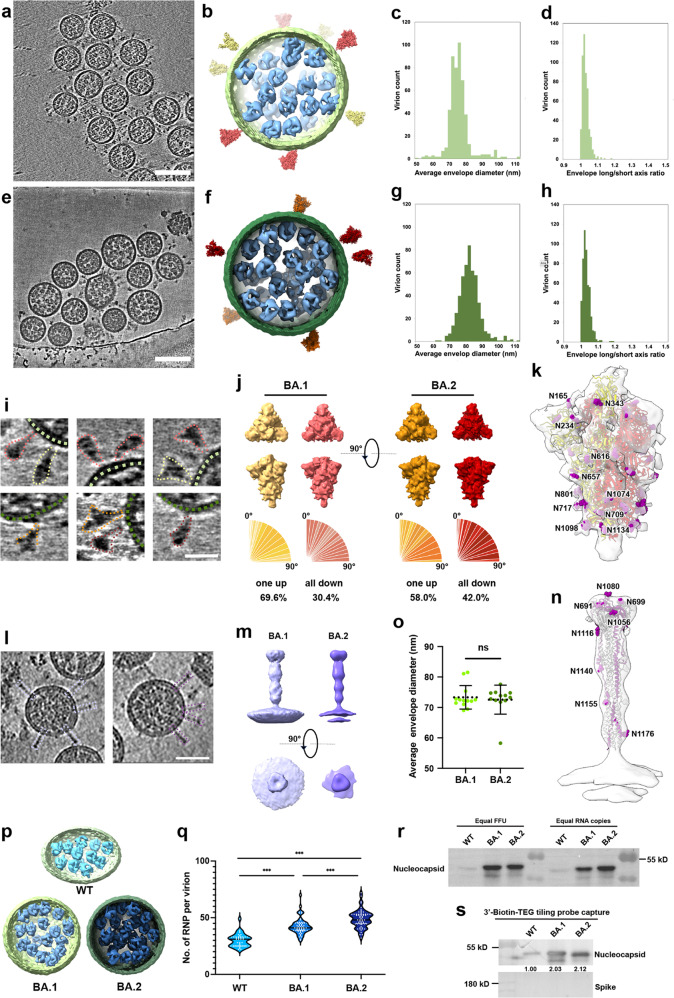
Cryo-electron tomography of Omicron lineages BA.1 and BA.2. Representative tomogram slice showing virions of BA.1 (**a**) and BA.2 (**e**), respectively; reconstructed models of both virions are shown in **b** and **f**, demonstrating the spikes (red and orange), the lipid envelope (green), and the RNPs (blue). Quantitative analysis of the BA.1 and BA.2, showing the envelope diameter (**c**, **g**) and the long/short axis ratio (**d**, **h**) measured from the corresponding tomograms. BA.1, *n* = 506; BA.2, *n* = 509. Scale bars: **a** and **e**, 100 nm. **i** Representative views of pre-fusion spikes, with the virion envelope marked by a green dash and spikes outlined by red (all-RBD-down) and yellow (one-RBD-up). Scale bar, 20 nm. **j** Refined density map of BA.1 and BA.2 pre-fusion spike in all-RBD-down and one-RBD-up conformations, showing both the side view and the top view, distributions of the spike tilt angle in two lineages. **k** BA.1 one-RBD-up map fitted with an atomic model of pre-fusion spike (PDB: 6VSB). Densities corresponding to N-linked glycans are highlighted in purple. **l** Representative views of post-fusion spikes, outlined by a purple dash. Scale bar, 50 nm. **m** Refined density maps by sub-tomogram averaging, showing the post-fusion spikes in BA.1 (left, resolution 26 Å) and BA.2 (right, resolution 28 Å). **n** BA.2 post-fusion map fitted with an atomic model of post-fusion spike (PDB: 6M3W), showing N-linked glycans. **o** Quantitative analysis of the envelope diameter of post-fusion virions. The mean diameter for postfusion-enriched virions is 73.3 ± 3.9 nm for BA.1 (*n* = 13) and 72.5 ± 4.8 nm for BA.2 (*n* = 12); ns, not significant. **p** Representative drawing models of BA.1 and BA.2, showing the RNPs (blue). **q** Quantitative analysis of RNPs per virion in three SARS-COV-2 variants. WT, *n* = 32; BA.1, *n* = 50; BA.2, *n* = 53; ***P < 0.001. **r** Immunoblotting analysis of the level of nucleocapsid protein in virus stock of WT, BA.1, and BA.2, with an equal amount of FFU (5000 FFU) or RNA copies (6000 copies) as indicated. **s** Immunoblotting analysis of biotin-modified oligo probe-based SARS-CoV-2 RNA pull-down enrichments

To obtain a higher resolution map of the viral spikes, we performed sub-tomogram averaging analysis. a total of 28,584 spikes were identified from BA.1 and 15,137 from BA.2 using template matching (Fig. 1i). Based on the template matching results, a mean number of 22.7 ± 7.7 pre-fusion spikes were found on each BA.1 virion and 35.3 ± 11.2 per BA.2 virion (Supplementary Fig. 1). This observation is comparable with the spike copy number (26 ± 11) reported in WT virion.^1^ After classification, two conformations of the pre-fusion spikes were resolved, the closed state with all RBDs “down” and the open state with only one RBD “up”. For BA.1 lineage, the density maps corresponding to RBD-down and one-RBD-up were refined to 8.3 Å and 9.1 Å resolution, respectively. While for the BA.2, the RBD-down and one-RBD-up conformation was at 7.3 Å and 8.3 Å (Fig. 1j and Supplementary Figs. 2 and 3). The proportion of the RBD-down conformation among all pre-fusion spikes was estimated to be 30.4% in BA.1 and 42.0% in BA.2 (Fig. 1j). Consistent with previous studies of the WT virions, orientation distribution analysis of the pre-fusion spikes showed that they swing freely around their stem, leading to poor resolution at the membrane-proximal stalk and missing densities at the transmembrane site (Fig. 1j). Several N-linked glycans are visible in both conformations (Supplementary Fig. 4), among which N616, N717, N801, N1098, and N1134 were best resolved, while N603 and N343 were poorly fitted (Fig. 1k).

The post-fusion spikes were rarely found on the virions. From 1752 virions, only 265 contain post-fusion spikes. Among them, 6% showed a high proportion of post-fusion spikes (Fig. 1l). Quantification analysis revealed a mean number of 3.0 post-fusion spikes per BA.1 virion and 2.6 per BA.2 virion (Supplementary Fig. 5). 102 and 110 post-fusion spikes were manually picked from BA.1 and BA.2 lineages, respectively. The final refined map reached a resolution of 26 Å and 28 Å (Fig. 1m and Supplementary Fig. 6) and was well-fitted with the previously reported structure (PDB: 6M3W). Densities corresponding to N-linked glycans are displayed in Fig. 1n. Among them, glycans around the stalk were well resolved, while the ones on the top were poorly fitted (Fig. 1n). The mean diameters of the post-fusion virions are 73.3 ± 3.9 nm for BA.1 and 72.5 ± 4.8 nm for BA.2 (Fig. 1o).

Compared to the WT, the lumen of both BA.1 and BA.2 lineages are densely packed with bucket-like densities (Fig. 1p), most likely representing ribonucleoproteins (RNPs). Interestingly, the mean number of RNPs increased from 30.0 ± 5.9 in WT to 43.4 ± 8.8 and 47.6 ± 7.8 in BA.1 and BA.2, respectively (Fig. 1q). To rigorously examine the abundance of nucleocapsids, an equal amount of focus forming units (FFUs) or RNA genome copies among WT, BA.1, and BA.2 were collected and subjected to Western Blot analysis. The immunoblotting results indicated that the level of nucleocapsid protein was approximately 1.9-2.2-fold higher in BA.1 and BA.2 than in WT (Fig. 1r). Moreover, SARS-CoV-2 RNA genome-pull down assays were further performed to determine the level of RNA genome-binding nucleocapsid during the life cycle of the viruses. We found a 2.12-fold nucleocapsid protein in BA.2 RNA-pull down enrichments than WT (Fig. 1s). By contrast, we observed a 2.03-fold higher nucleocapsid in BA.1 RNA-pull down than in WT (Fig. 1s). The 2-fold increase in RNA-protein binding affinity may be jointly contributed by the mutations in viral RNA and nucleocapsid protein.

Our cryo-ET study of the BA.1 and BA.2 subvariants provides the overall landscape and fine structural insight into Omicron. Sub-tomogram averaging analysis resolved the needle-like post-fusion spikes and the pre-fusion spikes in both open and close states. We did not observe dramatic structural changes in the prefusion spikes at nanoscopic scale between different variants (Supplementary Fig. 7). Virions inactivated with paraformaldehyde exhibited most of the spikes in pre-fusion conformation, different from those inactivated with β-propiolactone.^2^ However, a small percentage of virions also showed a high proportion of post-fusion spikes. Interestingly, these virions all have ambiguous RNPs, while the β-propiolactone-fixed virions show easily distinguishable RNP particles.

We report a significant increase in the number of RNPs in both BA.1 and BA.2 lineages. However, we did not observe any packing units of the RNPs. We propose that these RNP particles are highly heterogeneous and densely but randomly distributed inside viral lumen. Although they do not seem to form an ordered lattice beneath the membrane, the density of RNPs is higher at the membrane-proximal than the central zone. Given the spherical morphology of omicron virions, these membrane proximal RNPs may be linked to the virus envelope and increase membrane curvature.^3^ It has been reported that, when mixed with RNA, SARS-CoV-2 N protein can form gel-like condensates, structurally similar to RNPs. This phase separation behavior can provide a membrane-less compartment to concentrate and protect viral RNA.^4^ The formation of RNP condensates may also act as a rigid backbone that maintains the spherical shape of the virus envelope.

Previous studies have suggested a role of N protein in the immune evasion of SARS-CoV-2. After virus infection, the N protein can prevent the recognition and cleavage of viral dsRNA and suppress siRNA-induced RNA degradation. Moreover, the N protein can significantly elevate the replication of virus RNA and down-regulate genes that establish the host antiviral state (Supplementary Fig. 8).^5^ Our observation of up-regulated N protein in omicron variants may hint at their massive immune evasion, and suggest N protein as a promising diagnostic and therapeutic target against the SARS-CoV-2 epidemic.

## Supplementary information

Supplementary Information

Cryo-electron tomography of Omicron lineages BA.1

Cryo-electron tomography of Omicron lineages BA.2

## Data Availability

The density maps of the pre-fusion spike and post-fusion spike have been deposited in the Electron Microscopy Data Bank under accession codes EMD-35239, EMD-34339, EMD-34343, EMD-34340, EMD-34338, and EMD-34341.
