# Supplementary material for: Structural characteristics of the SARS-CoV-2 Omicron lineages BA.1 and BA.2 virions
Source: Signal Transduct Target Ther. 2023 Mar 20;8:131. doi: 10.1038/s41392-023-01385-9 (PMC10026235; doi:10.1038/s41392-023-01385-9)
Supplement: Supplementary file 1 — Supplementary Information [file 41392_2023_1385_MOESM1_ESM.docx]

**Supplementary Information**

**Structural** **Characteristics of the SARS-CoV-2 Omicron Lineages BA.1 and BA.2 Virions**

Xiaoyu Ma^1,6^, Yanqun Wang^2,6^, Yuanzhu Gao^1,6^, Yiliang Wang^2,6^, An Yan^1^, Jiantao Chen^2^, Lu Zhang^3^, Peiyi Wang^1^, Jincun Zhao^2,4,5^🖂 , and Zheng Liu^1^🖂

^1^Cryo-electron Microscopy Center, Southern University of Science and Technology, Shenzhen, Guangdong, China

^2^State Key Laboratory of Respiratory Disease, National Clinical Research Center for Respiratory Disease, Guangzhou Institute of Respiratory Health, the First Affiliated Hospital of Guangzhou Medical University, Guangzhou, Guangdong, China

^3^Health and Quarantine Laboratory, Guangzhou Customs District Technology Centre, Guangzhou, China

^4^Guangzhou Laboratory, Bio-Island, Guangzhou, China

^5^Institute of Infectious disease, Guangzhou Eighth People's Hospital of Guangzhou Medical University, Guangzhou, Guangdong, China

^6^These authors contributed equally

Correspondence: Zheng Liu ([liuz3@sustech.edu.cn](mailto:liuz3@sustech.edu.cn)) or Jincun Zhao ([zhaojincun@gird.cn](mailto:zhaojincun@gird.cn)).

**Supplementary Methods**

**Cell line and viruses**

Vero E6 cells (African green monkey kidney cells, ATCC C1008) were grown in Dulbecco’s modified Eagle’s medium (DMEM, Gibco) supplemented with 10% FBS. SARS-CoV-2 variants, including Omicron BA.1 (number: IQTC-IM2202090420) and BA.2 (number: IQTC-IM22003633), were isolated from COVID-19 patients, passaged, and titered on Vero E6 cells. Experiments related to authentic SARS-CoV-2 were conducted in Guangzhou Customs District Technology Center BSL-3 Laboratory.

**Virus proliferation and purification**

For the preparation and enrichment of virus samples, Omicron viruses (BA.1 and BA.2) were proliferated using Vero E6 cells in T75 culture flasks. Cells were infected with SARS-CoV-2 at a multiplicity of infection (MOI) of 0.02 for 1 hour and cultured under the indicated conditions of 37°C, 5% CO_2_. Two days later, 40 ml cell supernatant was cleared from cell debris at 3,500 g centrifugation for 20 min and inactivated with paraformaldehyde for 24 hours at 4°C. Then the fixed virions were pelleted by ultracentrifugation (Beckman Optima XPN-100, Beckman Coulter, Inc. Brea, CA) in SW41Ti rotor at 50,000 g for 3 hours at 4°C and resuspended in 20 μl PBS buffer for 1 hour^1^. The integrity and the abundance of virus were check with negative electron microscopy.

**Cryo-ET sample preparation and data acquisition**

The concentrated virus suspension was mixed with 6-nm gold fiducials (Electron Microscopy Sciences, Hatfield, PA) at a ratio of 5:1.4 μl mixture was applied onto a glow discharged copper grid coated with holey carbon film (Quantifoil R 2/1, 200 mesh), blotted for 4 s with force 1, then plunge-freezing into liquid ethane using a Vitrobot Mark IV system (humidity 98%, temperature 4°C, Thermo Fisher Scientific Inc. Waltham, MA).

The cryo grids were loaded into a Titan Krios G3 transmission electron microscope (Thermo Fisher Scientific Inc) operated at a voltage of 300kV. Images were recorded on a K2 Summit direct detection camera (Gatan, Inc. Pleasanton, CA) in super-resolution mode with a Gatan Quantum energy filter (GIF Quantum) in zero-loss mode with a slit width of 20eV. Tilt series were acquired with SerialEM using a dose symmetric scheme with 3° increment step from -60° to +60° ^2^. The detailed imaging parameters are listed in **Supplementary Table 1**.

**Image processing**

Collected images were motion-corrected using MotionCor2^3^. Cryo tomogram reconstruction was performed semi-automatically using the batch processing function of the IMOD software package^2,4^. Sub-volumes of virions were cropped from each tomogram and particle picking was performed using template matching with emClarity^5^. A low-resolution map generated from PDB 7WK5 was use as template for pre-fusion S spikes and EMD-30429 for post-fusion. Selected particles were imported into Relion-4.0 for classification and sub-tomogram averaging^6^. The orientation and the distribution of the spikes was measured using in-house scripts. Atomic models (PDB 6VSB and PDB 6M3W) of the pre- and post-fusion S was fitted to the corresponding density map with the Fit in Map tool in UCSF Chimera^7^. For drawings of viral lumen, 405 RNPs were manually picked and averaged with eman2. The low-resolution density map of RNP were fitted back to the tomogram with ChimeraX, to demonstrate the locations of RNPs *in situ*.

**Quantification and Statistic analysis**

The diameter of virions was measured with the Feret’s diameter function in FIJI^8^. The number of RNPs was manually counted and plotted with Prism (GraphPad Software, San Diego, CA).

**SARS-CoV-2 RNA genome pull-down**

The probes targeting SARS-CoV-2 overlapped sequences among different strains (WT, BA.1, and BA.2) were designed online (https://www.biosearchtech.com/stellaris). Detailed information regarding full probe sequences is available in **Supplementary Table 2**. Oligos probes with 3’ biotin-TEG modifications were synthesized at Tsingke Biotechnology Co., Ltd. SARS-CoV-2 RNA pull-down was performed similarly as described in our prior publications with minor modifications^9^. Briefly, 1 mL IP Lysis Buffer (Beyotime, # P0013) containing 1 mM PMSF, 10 mM Ribonucleoside Vanadyl Complex (RVC) (NEB, #S1402S), and a proteasome inhibitor cocktail (Beyotime, # P1008) was added to each plate with 2.0 × 106 SARS-CoV-2-infected Vero-E6 cells and incubated at 4°C for 5 min. Subsequently, the lysates were centrifuged at 12,000 g for 10 min at 4 °C and 10 μL of 100 mM tilling Oligo Probe Pools were added per mL of supernatants. Afterward, 1 mL of the supernatant containing tiling probes was transferred to MagStrep XT Beads (IBA, # 2-4090-002) and incubated overnight at 4 °C with rotation. Next, the beads were washed with IP Lysis Buffer (Beyotime, # P0013) containing 1 mM PMSF, 10 mM Ribonucleoside Vanadyl Complex (RVC) (NEB, #S1402S),and a proteasome inhibitor cocktail (Beyotime, # P1008) five times. The beads-coupled proteins were obtained by adding 1*SDS-loading buffer and then heated at 100°C for 10 min. The final elution samples were subjected to Western Blot analysis.

**Western Blot analysis**

To compare the abundance of Nucleocapsid protein between WT, BA.1, and BA.2, the virus stock containing an equal amount of focus forming units (5,000 FFU) or RNA copies (6,000 copies) among different stains was mixed with a corresponding volume of 5*SDS*loading buffer to prepare the samples subjected to Western Blot analysis. Then, the samples were heated at 99°C for 10 min and loaded on an SDS-polyacrylamide electrophoresis gel. Proteins were transferred to the PVDF membrane by wet transfer in 1x Tris-Glycine buffer containing 20% methanol. Membranes were blocked in 5% skim milk and then probed with QuickBlockTM Primary Antibody Dilution Buffer (Beyotime, Cat#P0256) containing indicated primary antibodies against virion protein. The band signals were visualized with enhanced chemiluminescence (Thermo Fisher Scientific, MA, USA, Cat#34580) and imaged by a 5200-image analysis system (Tannon, Shanghai, China).

**Supplementary Table 1. Cryo-EM and image-analysis specifics**

| EM grids | Quantifoil R2/1, 200 mesh |
| --- | --- |
| Microscope | Titan Krios G3 (D3706) |
| Energy | 300 keV |
| Camera: recording mode | Gatan K3 summit: superresolution |
| Tomography software | SerialEM |
| Calibrated pixel size | 0.665 |
| Contrast mechanism | Volta phase contrast & defocus phase contrast |
| Defocus (nominal) | -0.5 (Volta) or -3 to -5 µm (defocus) |
| Cumulative dose | 120 e^-^ / Å^2^ |
| Tilt range | ±60°, dose-symmetric |
| Tilt increment | 3° |
| Tomogram processing | IMOD 4.10 |
| Template matching | emClarity |
| Subtomogram analysis | RELION 4.0 |
| Tomogram visualization | UCSF ChimeraX, IMOD 4.10 |
| Calculations | Prism 9, FIJI |
| Figure/movie editing | Adobe Photoshop, iMovie |

**Supplementary Table 2. Sequence of SARS-CoV-2 3’-Biotin-TEG tiling probe**

| **SARS-CoV-2 3’-Biotin-TEG tiling probe- set** | | **Numbers** | | **NumProbe (5'-> 3')** | | **Probe position *** | | **Percent GC** | |
| --- | --- | --- | --- | --- | --- | --- | --- | --- | --- |
| #1 | | 1 | | taaggatcagtgccaagctc | | 10 | | 50.00% | |
|  | | 2 | | atcgacatagcgagtgtatg | | 110 | | 45.00% | |
|  | | 3 | | agtccagttgttcggacaaa | | 210 | | 45.00% | |
|  | | 4 | | aggtgtctgcaattcatagc | | 311 | | 45.00% | |
|  | | 5 | | cccttggttgaatagtcttg | | 411 | | 45.00% | |
|  | | 6 | | tcacacttcatgagagttga | | 520 | | 40.00% | |
|  | | 7 | | agtaaccacaagtagtggca | | 627 | | 45.00% | |
|  | | 8 | | tccaaaggcaatagtgcgac | | 770 | | 50.00% | |
|  | | 9 | | cattaagaccttcggaacct | | 891 | | 45.00% | |
|  | | 10 | | caaacctttcacagtttcca | | 1031 | | 40.00% | |
|  | | 11 | | agaggactcagtattgattt | | 1147 | | 35.00% | |
|  | | 12 | | tagcggccttctgtaaaaca | | 1248 | | 45.00% | |
|  | | 13 | | gtaggccattacaactagat | | 1349 | | 40.00% | |
|  | | 14 | | tctcttcaagccaatcaagg | | 1449 | | 45.00% | |
|  | | 15 | | acaggtgacaatttgtccac | | 1550 | | 45.00% | |
|  | | 16 | | ttaagtttagctccaccaat | | 1651 | | 35.00% | |
|  | | 17 | | ttttagaggcatgagtaggc | | 1751 | | 45.00% | |
|  | | 18 | | cttcactagtaggttgttct | | 1866 | | 40.00% | |
|  | | 19 | | atattaggtgcaagggcaca | | 1966 | | 45.00% | |
|  | | 20 | | tcacactcttgtaaccttgc | | 2067 | | 45.00% | |
| #2 | | 1 | | gcatttctcgcaaattccaa | | 58 | | 40.00% | |
|  | | 2 | | agcaccataatcaaccacac | | 183 | | 45.00% | |
|  | | 3 | | tgtgttacatagccaagtgg | | 287 | | 45.00% | |
|  | | 4 | | tgttcttcaggtgttttaga | | 422 | | 35.00% | |
|  | | 5 | | acacttttatcacctctctt | | 527 | | 35.00% | |
|  | | 6 | | accttaatagtcctcacttc | | 632 | | 40.00% | |
|  | | 7 | | acatcagctccatccaaata | | 734 | | 40.00% | |
|  | | 8 | | gggtatttccactttttagt | | 899 | | 35.00% | |
|  | | 9 | | cttgtagagcaggtggatta | | 1012 | | 45.00% | |
| #3 | | 1 | | aagcagcggttgagtagatt | | 39 | | 45.00% | |
|  | | 2 | | ccagtacagtaggttgcaat | | 142 | | 45.00% | |
|  | | 3 | | aattgcatgattgcagccaa | | 337 | | 40.00% | |
|  | | 4 | | gtacattctaaccatagctg | | 443 | | 40.00% | |
|  | | 5 | | gctctattacgtttgtaaca | | 544 | | 35.00% | |
|  | | 6 | | attccaattgtgtagtttgc | | 644 | | 35.00% | |
|  | | 7 | | acgatgtaagaagactggtc | | 763 | | 45.00% | |
|  | | 8 | | ttagctctcaggttgtctaa | | 880 | | 40.00% | |
|  | | 9 | | cagacactaatgcctgatct | | 1026 | | 45.00% | |
|  | | 10 | | ttctttgcaagttcagcttc | | 1159 | | 40.00% | |
|  | | 11 | | actatcgccagtaacttcta | | 1298 | | 40.00% | |
|  | | 12 | | ttgtgactttttgctacctg | | 1414 | | 40.00% | |
| #4 | | 1 | | ttcttgatccatattggctt | | 13 | | 35.00% | |
|  | | 2 | | ggtatttgtacatacttacc | | 114 | | 35.00% | |
|  | | 3 | | ttcgcggagttgatcacaac | | 220 | | 50.00% | |
|  | | 4 | | tacgacatcagtactagtgc | | 324 | | 45.00% | |
|  | | 5 | | aattgtcatcttcgtccttt | | 424 | | 35.00% | |
|  | | 6 | | tgtttagcaacagctggaca | | 527 | | 45.00% | |
|  | | 7 | | gccttaaagcatagacgagg | | 628 | | 50.00% | |
|  | | 8 | | acaaaatcataccagtcctt | | 728 | | 35.00% | |
|  | | 9 | | caataccagcatttcgcatg | | 835 | | 45.00% | |
|  | | 10 | | tctacaacaggaactccact | | 935 | | 45.00% | |
|  | | 11 | | cccacttaatgtaaggcttt | | 1036 | | 40.00% | |
|  | | 12 | | tgcagaatgcatctgtcatc | | 1157 | | 45.00% | |
| #5 | | 1 | | cacaacagcatcgtcagaga | | 3 | | 50.00% | |
|  | | 2 | | cagtctcagtccaacatttt | | 121 | | 40.00% | |
|  | | 3 | | cctaggattcttgatggatc | | 227 | | 45.00% | |
|  | | 4 | | gcatactcctgattaggatg | | 344 | | 45.00% | |
|  | | 5 | | cccaataccttgaagtgtta | | 460 | | 40.00% | |
|  | | 6 | | tatgcaagcaccacatctta | | 567 | | 40.00% | |
|  | | 7 | | cctggagcattgcaaacata | | 668 | | 45.00% | |
|  | | 8 | | aacttgtccattagcacaca | | 774 | | 40.00% | |
|  | | 9 | | gtctttcagtacaggtgtta | | 895 | | 40.00% | |
|  | | 10 | | aattctctgtcagacagcac | | 995 | | 45.00% | |
|  | | 11 | | tttcaaaggtgtactctcct | | 1111 | | 40.00% | |
|  | | 12 | | ggtgcacttaatggcattac | | 1220 | | 45.00% | |
|  | | 13 | | tttgcataccaaccttttga | | 1333 | | 35.00% | |
|  | | 14 | | gcaagctgtatacactatgc | | 1434 | | 45.00% | |
|  | | 15 | | aacactctacacgagcacgt | | 1534 | | 50.00% | |
|  | | 16 | | tctggcattgacaacactca | | 1677 | | 45.00% | |
| #6 | | 1 | | ccttaggtatgccaggtatg | | 1 | | 50.00% | |
|  | | 2 | | tcttatagcttcttcgcggg | | 102 | | 50.00% | |
|  | | 3 | | cacctgtagaaaaacctagc | | 202 | | 45.00% | |
|  | | 4 | | tgtttaaattgatctccagg | | 302 | | 35.00% | |
|  | | 5 | | gactctgtcagagagatttt | | 402 | | 40.00% | |
|  | | 6 | | tggcacgtctatcacataga | | 502 | | 45.00% | |
|  | | 7 | | ccccattgttgaacatcaat | | 602 | | 40.00% | |
|  | | 8 | | agctagacacctagtcatga | | 702 | | 45.00% | |
|  | | 9 | | tgtgttgaacctttctacaa | | 802 | | 35.00% | |
|  | | 10 | | cattctacatcagcttgagg | | 902 | | 45.00% | |
|  | | 11 | | tacaccatctgtgaatttgt | | 1002 | | 35.00% | |
|  | | 12 | | cacaaccaggcaagttaagg | | 1102 | | 50.00% | |
|  | | 13 | | actcacatggactgtcagag | | 1219 | | 50.00% | |
|  | | 14 | | gcatgatgtctacagacagc | | 1322 | | 50.00% | |
|  | | 15 | | agtgtcccttatttacaaca | | 1483 | | 35.00% | |
|  | | 16 | | tactggtttaatgttgcgct | | 1638 | | 40.00% | |
|  | | 17 | | cttggctatgtcagtcatag | | 1767 | | 45.00% | |
| #7 | | 1 | | gcacgtacatgtcttatagc | | 115 | | 45.00% | |
|  | | 2 | | gtaggtacagcaactaggtt | | 226 | | 45.00% | |
|  | | 3 | | gcactacattccaaggaagt | | 348 | | 45.00% | |
|  | | 4 | | ctcaggtcctattttcacaa | | 473 | | 40.00% | |
|  | | 5 | | tgcattaccatggacttgac | | 662 | | 45.00% | |
|  | | 6 | | acaagccgcattaatcttca | | 788 | | 40.00% | |
|  | | 7 | | ctacatcagcttgaggtaca | | 900 | | 45.00% | |
|  | | 8 | | acaaccaggcaagttaaggt | | 1103 | | 45.00% | |
|  | | 9 | | cttgttttccatgagactca | | 1236 | | 40.00% | |
|  | | 10 | | tgtttgtaaacccacaagct | | 1399 | | 40.00% | |
|  | | 11 | | tactggtttaatgttgcgct | | 1640 | | 40.00% | |
|  | | 12 | | cttggctatgtcagtcatag | | 1769 | | 45.00% | |
| #8 | | 1 | | ttgaccatcaactctaccat | | 37 | | 40.00% | |
|  | | 2 | | tttttacggcttctccaatt | | 167 | | 35.00% | |
|  | | 3 | | attcatccatagctaattct | | 308 | | 30.00% | |
|  | | 4 | | ctagtccaatcagtagatgt | | 410 | | 40.00% | |
|  | | 5 | | gatgaacctgtttgcgcatc | | 510 | | 50.00% | |
|  | | 6 | | agtcactttgacaaccttag | | 610 | | 40.00% | |
|  | | 7 | | agattaggcatagcaacacc | | 726 | | 45.00% | |
|  | | 8 | | gttgagtatattttgcgaca | | 833 | | 35.00% | |
|  | | 9 | | aaaacagctgtacctggtgc | | 939 | | 50.00% | |
|  | | 10 | | tgtacagttgcacaatcacc | | 1041 | | 45.00% | |
|  | | 11 | | gcttttgttgtataaaccca | | 1166 | | 35.00% | |
|  | | 12 | | taacaaaggctgtccaccat | | 1268 | | 45.00% | |
|  | | 13 | | gtaatttgcatgcatgacat | | 1369 | | 35.00% | |
|  | | 14 | | aagacataacagcagtaccc | | 1469 | | 45.00% | |
|  | | 15 | | cacactgactagagactagt | | 1635 | | 45.00% | |

**
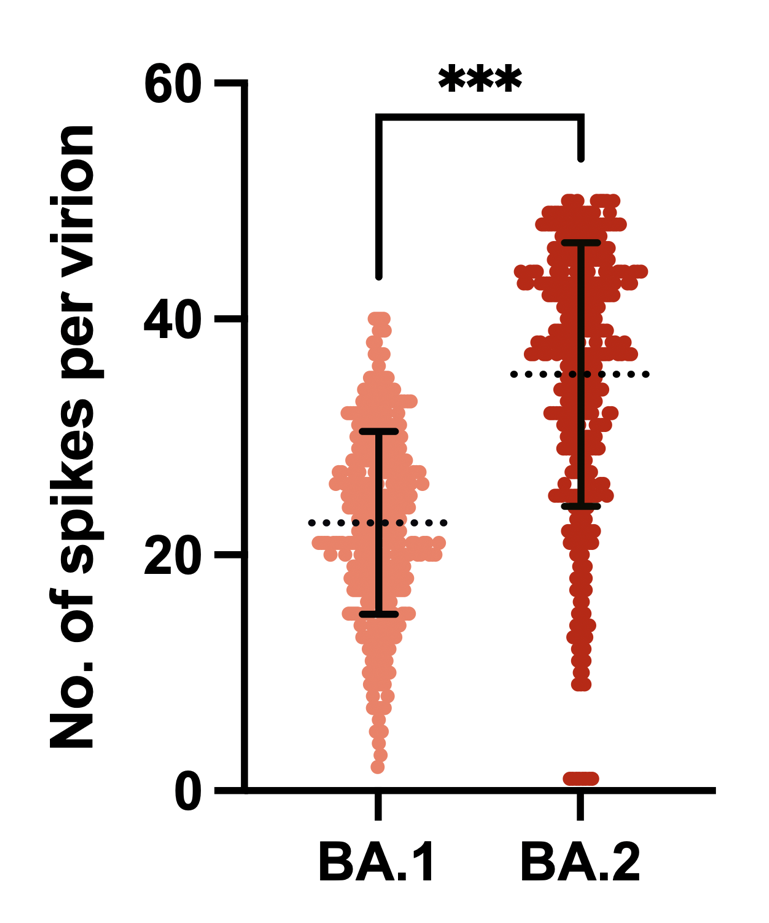
**

**Supplementary Figure 1. Quantitative analysis of pre-fusion spikes per virion in BA.1 and BA.2 lineages.**

The number of all pre-fusion spikes is 22.7 ± 7.7 (mean ± SD) in BA.1 (*n* = 402) and 35.3 ± 11.2 in BA.2 (*n* = 407). Mean and standard deviation range are indicated, ***, *P*<0.001. Considering the high rate of false positives from template-matching method, the true number of pre-fusion spikes should be smaller than our measurements.

**Supplementary Figure 2. Fourier-shell-correlation (FSC) plot of BA.1 pref-usion spike sub-tomogram averaged density maps. Related to Figure 1j.**

**Supplementary Figure 3. FSC plot of BA.2 pre-fusion spike sub-tomogram averaged density maps. Related to Figure 1j.**

**
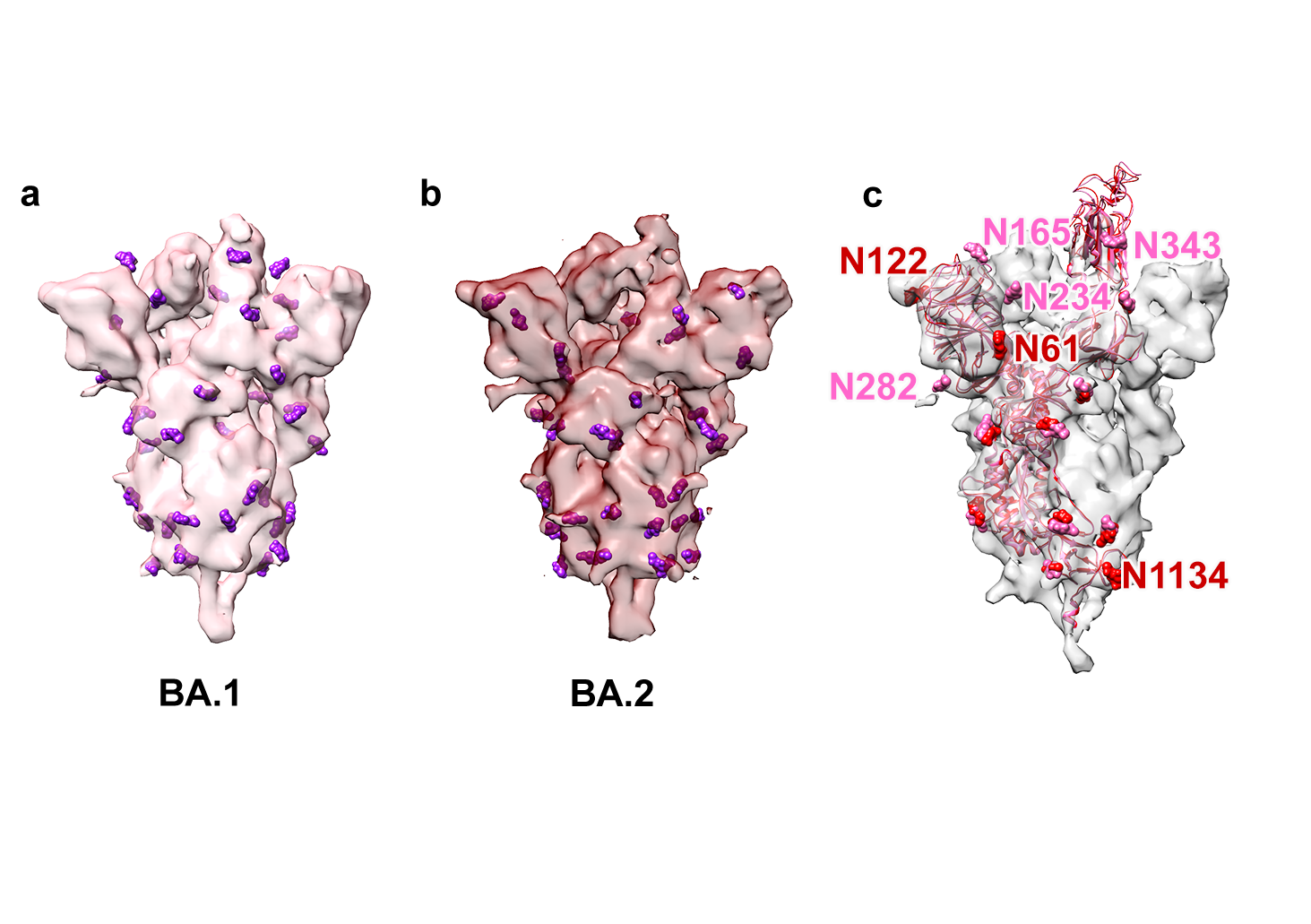
**

**Supplementary Figure 4. Glycans sites for BA.1 and BA.2.**

(**a**) and (**b**) PDB 7XO5 (BA.1) and PDB 7XOA were fixed into corresponding density maps, demonstrating the glycans sites (purple). (**c**) comparison of glycans sites of one protomer in two variants. Unique glycans sites are mark out for BA.1 (pink) and BA.2 (red).

**
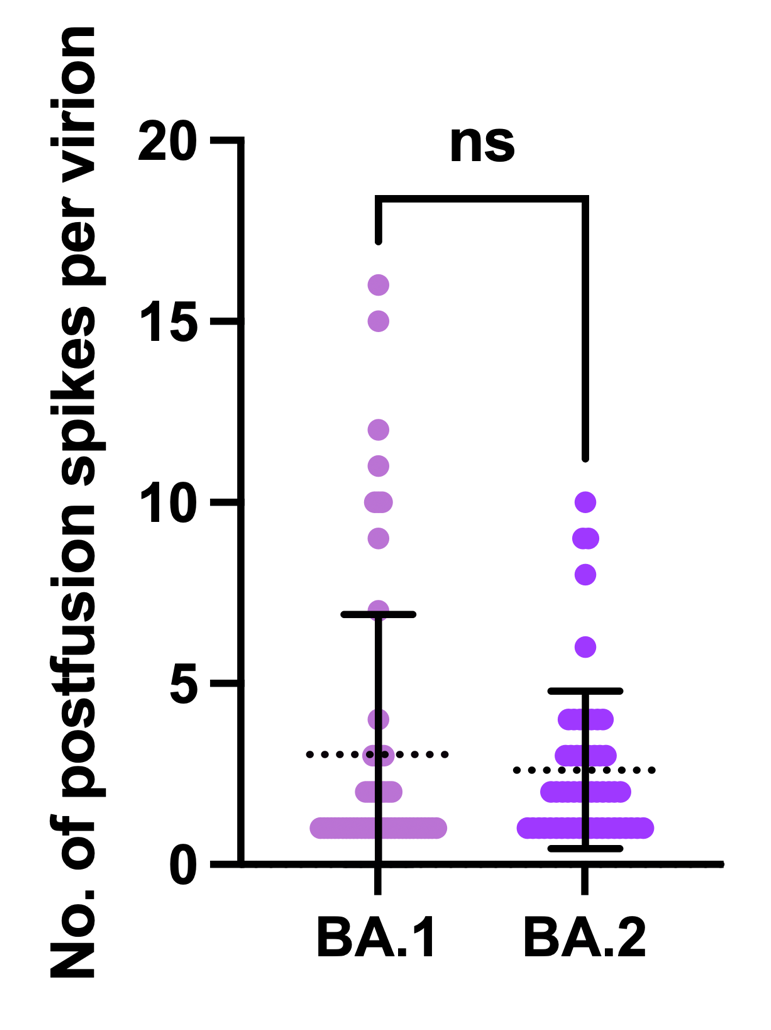
**

**Supplementary Figure 5. Quantitative analysis of post-fusion spikes per virion in BA.1 and BA.2 lineages.**

The number of post-fusion spikes is 3.0 ± 3.9 in BA.1 (*n* = 54) and 2.6 ± 2.2 in BA.2 (*n* = 54); ns, not significant.

**Supplementary Figure 6. FSC plot of postfusion spike subtomogram averaged density maps. Related to Figure 1m.**

**
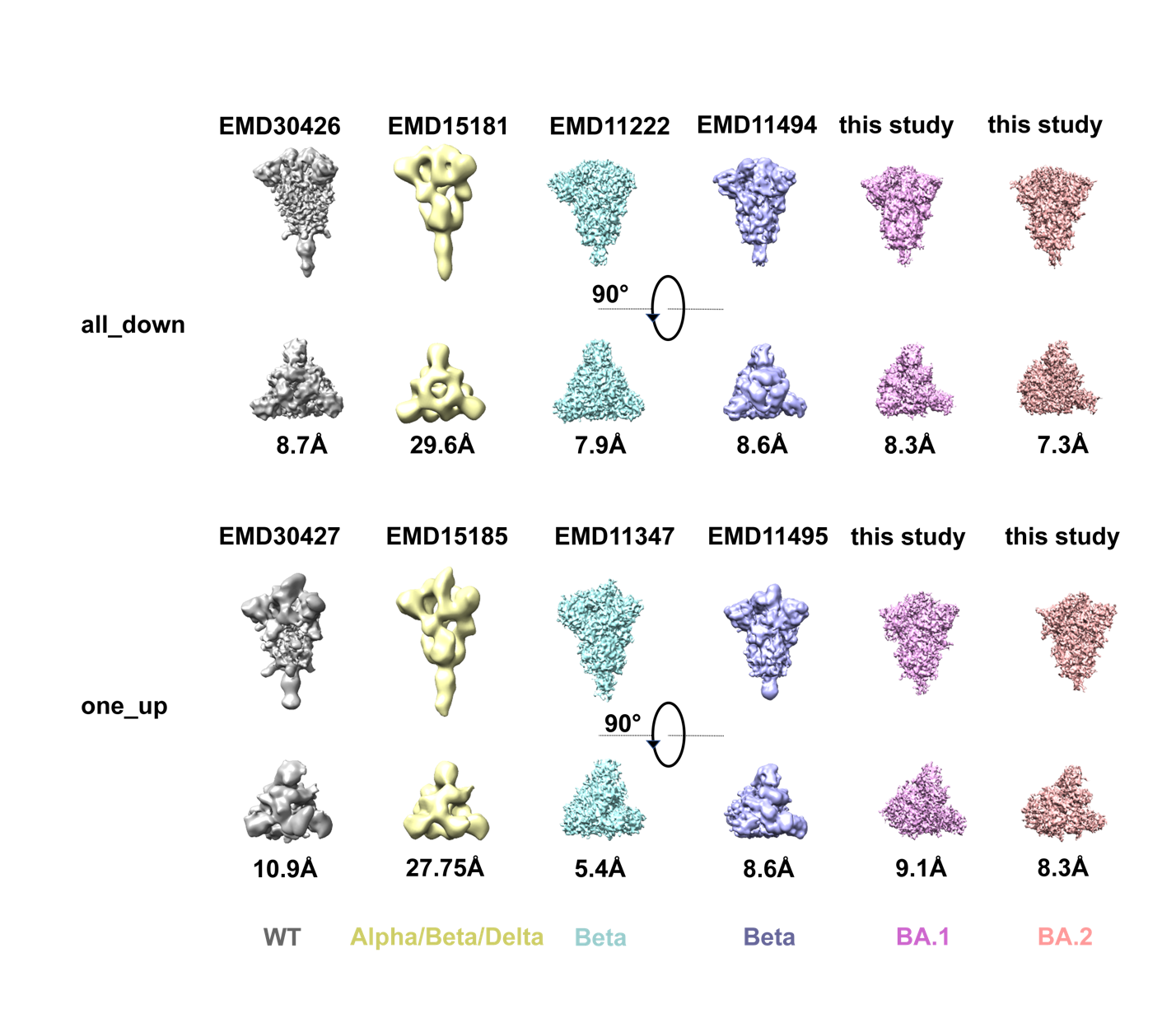
**

**Supplementary Figure 7. Structural comparison of pre-fusion spikes from different variants, all resolved by sub-tomogram averaging analysis.**

The two dominant conformations, all-RBD-down and one-RBD-up, are compared. Density maps from previous studies are downloaded from Electron Microscopy Data Bank (<https://www.ebi.ac.uk/emdb/>).


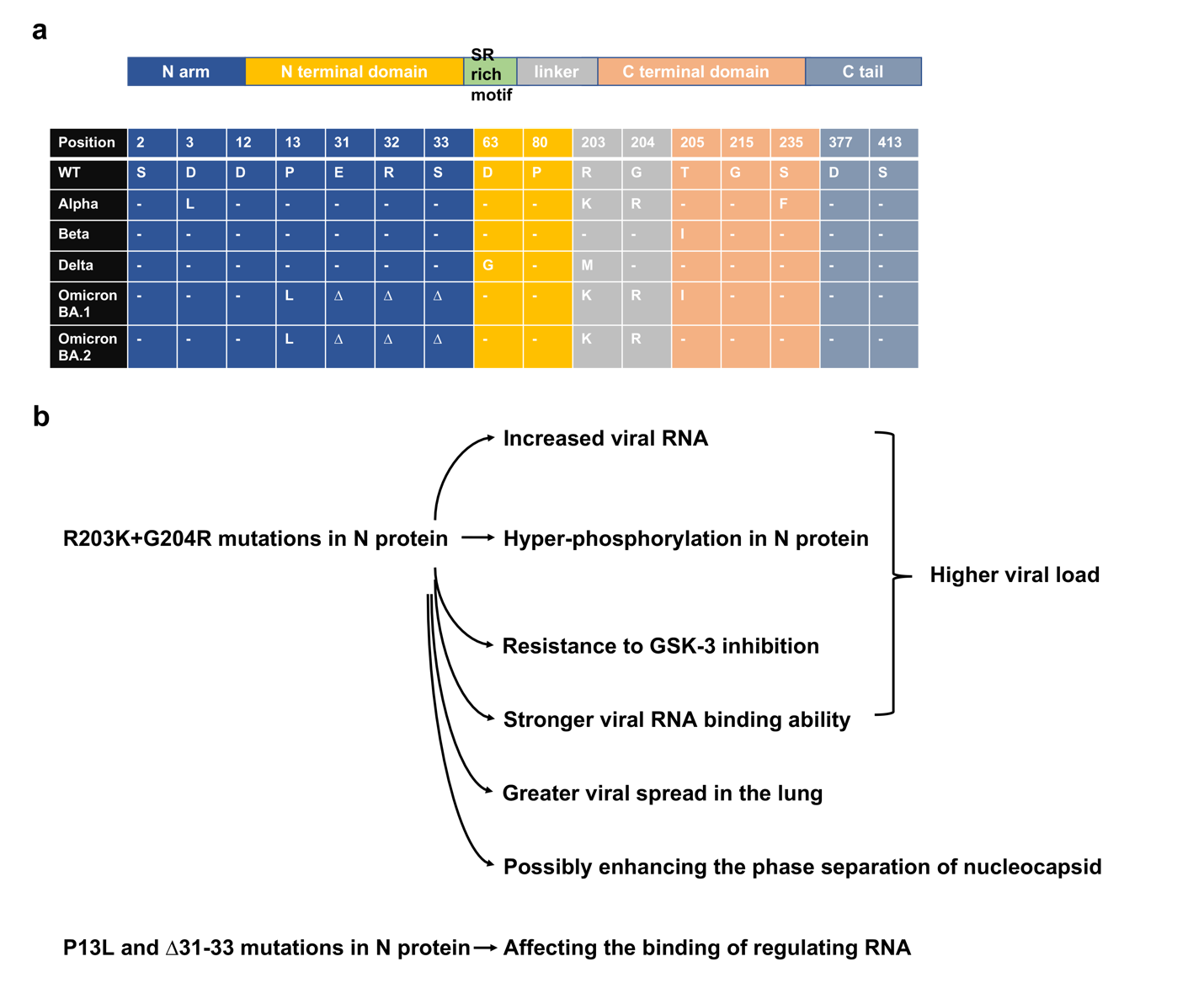


**Supplementary Figure 8. Unique mutations in Omicron N protein.**

(**a**) schematic diagram of the SARS-CoV-2 N protein modular organization, listing the unique mutations in different variants. (**b**) functions of the unique mutations in omicron variants reported by previous studies.^10,11^

**References**

1 Neuman, B. W., Adair, B. D., Yeager, M. & Buchmeier, M. J. Purification and electron cryomicroscopy of coronavirus particles. *Methods Mol Biol* **454**, 129-136 (2008).

2 Mastronarde, D. N. & Held, S. R. Automated tilt series alignment and tomographic reconstruction in IMOD. *J Struct Biol* **197**, 102-113 (2017).

3 Zheng, S. Q. *et al.* MotionCor2: anisotropic correction of beam-induced motion for improved cryo-electron microscopy. *Nat Methods* **14**, 331-332 (2017).

4 Kremer, J. R., Mastronarde, D. N. & McIntosh, J. R. Computer visualization of three-dimensional image data using IMOD. *J Struct Biol* **116**, 71-76 (1996).

5 Himes, B. A. & Zhang, P. emClarity: software for high-resolution cryo-electron tomography and subtomogram averaging. *Nat Methods* **15**, 955-961 (2018).

6 Kimanius, D., Dong, L., Sharov, G., Nakane, T. & Scheres, S. H. W. New tools for automated cryo-EM single-particle analysis in RELION-4.0. *Biochem J* **478**, 4169-4185 (2021).

7 Pettersen, E. F. *et al.* UCSF Chimera--a visualization system for exploratory research and analysis. *J Comput Chem* **25**, 1605-1612 (2004).

8 Schindelin, J. *et al.* Fiji: an open-source platform for biological-image analysis. *Nat Methods* **9**, 676-682 (2012).

9 Wang, Y. *et al.* A novel lncRNA linc-AhRA negatively regulates innate antiviral response in murine microglia upon neurotropic herpesvirus infection. *Theranostics* **11**, 9623-9651 (2021).

10 Johnson, B. A. *et al.* Nucleocapsid mutations in SARS-CoV-2 augment replication and pathogenesis. *PLoS Pathog* **18**, e1010627 (2022).

11 Mourier, T. *et al.* SARS-CoV-2 genomes from Saudi Arabia implicate nucleocapsid mutations in host response and increased viral load. *Nat Commun* **13**, 601 (2022).
